# Cytogenetic and pollen identification of genus *Gagnepainia* (Zingiberaceae) in Thailand

**DOI:** 10.3897/CompCytogen.v14i1.47346

**Published:** 2020-01-13

**Authors:** Paramet Moonkaew, Nattapon Nopporncharoenkul, Thaya Jenjittikul, Puangpaka Umpunjun

**Affiliations:** 1 Department of Plant Science, Faculty of Science, Mahidol University, Rama VI Road, Ratchathewi, Bangkok, 10400, Thailand Mahidol University Bangkok Thailand

**Keywords:** Chromosome number, Globbeae, *
Hemiorchis
*, meiotic figure, nuclear DNA content, palynology

## Abstract

*Gagnepainia godefroyi* K. Schumann, 1904 and *G. harmandii* K. Schumann, 1904 belong to the genus *Gagnepainia* K. Schumann, 1904 of the Ginger family. They have the potential to be developed as medicinal and attractive ornamental plants. To date, the knowledge on the cytological and reproductive aspects of *Gagnepainia* have not been publicly available. Therefore, the aims of this research are to investigate the cytogenetic and pollen characters of *Gagnepainia* species using light, fluorescence, and scanning electron microscopes. The regular meiotic figures of 15 bivalents are found in both species and presented for the first time. These evidences indicate that *Gagnepainia* is diploid and contains 2*n* = 2*x* = 30 with basic number of *x* = 15. The mean nuclear DNA contents range from 1.986 pg in *Gagnepainia* sp., 2.090 pg in *G. godefroyi* to 2.195 pg in *G. harmandii*. Pollens of all species are monad, inaperturate, prolate with bilateral symmetry, and thick wall with fossulate exine sculpturing. The pollen size of *G. harmandii* (74.506 ± 5.075 μm, 56.082 ± 6.459 μm) is significantly larger than that of *G. godefroyi* (59.968 ± 3.484 μm, 45.439 ± 2.870 μm). Both 2C DNA content and pollen size are the effective characteristics for species discrimination. The reproductive evidence of high meiotic stability and normal pollen production indicate that both *Gagnepainia* species have high fertility and seed productivity, which are in accordance with the broad distribution. The present study provides good cytogenetic and pollen characters not only for plant identification, but also plant fertility assessment through plant genetic resource management and improvement of *Gagnepainia*.

## Introduction

*Gagnepainia* K. Schumann, 1904 is a small tropical ginger genus belonging to tribe Globbeae of Zingiberaceae. It was taxonomically classified into the genus *Hemiorchis* Kurz, 1873, another member within the same tribe. According to taxonomic revision of Schumann (1904), it was separated from *Hemiorchis* and formally placed in a new genus, which was named in honour of François Gagnepain, a French botanist (1866–1952). Despite the fact that *Gagnepainia* is able to phylogenetically form a monophyletic clade with *Hemiorchis*, they are sister genera to each other (Williams et al. 2004, Pospíšilová et al. 2016).

*Gagnepainia* is a small, deciduous, perennial ginger which has a strong dormancy during the dry period. It has distinctive swollen rhizomes jointed with the base of well-developed pseudostems. The inflorescences consist of the numerous tiny butterfly-like flowers, usually emerging directly from the ground before the emergence of the leafy shoots. Remarkably, the trilobed labellum with a peg-shaped central lobe is a unique characteristic differentiating *Gagnepainia* from closely related genera, especially *Globba* Linnaeus, 1771 and *Hemiorchis* (Leong-Škorničková and Newman 2015). Currently, *Gagnepainia* comprises only two species, which can be characterized by lateral staminode shape and flower colour. *Gagnepainia godefroyi* K. Schumann, 1904 has creamy-white to pale orange flowers with broadly elliptic to obovate lateral staminodes. On the other hand, *G. harmandii* (Baill.) K. Schumann, 1904 has bright green flowers with oblanceolate lateral staminodes (Newman and Simon 2017). Geographically speaking, this genus is widely distributed across the Indo-Chinese and Indo-Burmese areas and Thailand, except the peninsular region (Larsen and Larsen 2006, Leong-Škorničková and Newman 2015). The species of *Gagnepainia* have the ethnomedical potential use for wound healing, inflammation treatment, and hemostasis. Because of their gorgeous flowers, both two species are also cultivated as ornamental plants (Chuakul and Boonpleng 2004, Prathanturarug et al. 2007).

Even though extensive studies on species belonging to the ginger family have been cytogenetically conducted, only chromosome numbers of *Globba* were reported for Globbeae. The species in genus *Globba* contain the diverse chromosome numbers of 2*n* = 20, 22, 24, 28, 32, 34, 48, 64, and 96. However, *x* = 8 is considered as the primary basic chromosome number (Mahanty 1970, Lim 1972, Takano 2001, Jatoi et al. 2007, Pospíšilová et al. 2016). In the sister genus *Hemiorchis*, only the chromosome number of *H. burmanica* Kurz has been reported as 2*n* = 30 (Pospíšilová et al. 2016).

It is evident that, to date, knowledge on the cytological and reproductive aspects have not publicly been available for the genus *Gagnepainia*. Therefore, the aims of this research are to intensively investigate the cytogenetic characters, including chromosome numbers, meiotic figures, and genome sizes (2C-value), of the genus *Gagnepainia* in Thailand. Pollen morphological study, using light (LM) and scanning electron (SEM) microscopes, were also conducted for the fertility assessment through further genetic resource conservation management and utilization of this genus.

## Materials and methods

### Sample collection and plant identification

**Figure 1.**
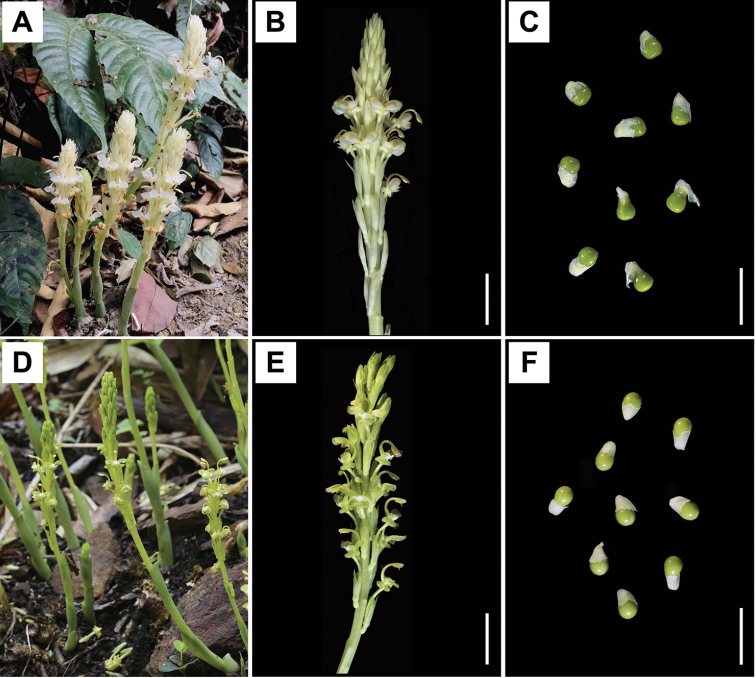
*Gagnepainia* spp. in Thailand. **A–C***G. godefroyi***A** inflorescences in habitat (PMNN022) **B** detail of inflorescences (PMNN011) **C** seeds (PMNN011) **D–F***G. harmandii***D** inflorescences in habitat (PMNN006) **E** detail of inflorescences (PMNN006) **F** seeds (PMNN006). Scale bars: 2 cm (**B, E**); 1 cm (**C, F**). Photo by N. Nopporncharoenkul.

We compiled a total of 19 accessions of *G. godefroyi* and *G. harmandii* in the present study. The accession number of each sample was assigned as PMNN (P. Moonkaew and N. Nopporncharoenkul) and followed by the reference number which referred to the population in district range. The majority of sample collections are from natural habitats in various parts of Thailand, whilst others (PMNN024, 025, 027, 028, and 030) were collected from the Queen Sirikit Botanic Gardens (QSBG), Chiang Mai, Thailand. The list of plant materials with their geographic localities are shown in Table 1. All samples were identified based on the floral characters which were described by Leong-Škorničková and Newman (2015). Representative flowers of each species are shown in Figure 1. Only one accession, PMNN021 from Khong Chiam, Ubon Ratchathani, has not flowered in either natural habitat or cultivation, so we assigned this unknown taxon as *Gagnepainia* sp. Plant samples have been meticulously preserved at Department of Plant Science, Faculty of Science, Mahidol University, Bangkok and QSBG, Chiang Mai, Thailand. The voucher specimens of all accessions are kept, in 70% ethanol, at the BKF and QBG herbaria.

**Table 1.** Cytogenetic and palynological characters of *Gagnepainia* accessions analyzed in the present study.

**Species**	**Locality**	**Accession no.**	**2*n***	***n***	**2C value (pg) ± S.E.**	**Mean 2C value (pg) ± S.E.**	**Ploidy level**	**Pollen size (µm)**			
								**Polar axis ± S.E.**	**Mean polar axis ± S.E.**	**Equatorial axis ± S.E.**	**Mean equatorial axis ± S.E.**
*Gagnepainia godefroyi* (Baill.) K. Schum.	Chiang Dao, Chiang Mai	PMNN030			2.133 ± 0.021	2.090 ± 0.028	2×		59.968 ± 3.484		45.439 ± 2.870
	Dan Sai, Loei	PMNN027			2.134 ± 0.004		2×				
	Erawan, Loei	PMNN029			2.067 ± 0.016		2×				
	Mae Ramat, Tak	PMNN020			2.059 ± 0.025		2×	61.875 ± 3.551		43.274 ± 1.936	
	Mae Rim, Chiang Mai	PMNN011	30	15II	2.090 ± 0.017		2×	59.427 ± 2.953		45.681 ± 2.185	
	Mae Sot, Tak	PMNN026	30^a^	15II	2.090 ± 0.006		2×	60.740 ± 3.915		45.142 ± 2.764	
	Pha Lat, Mueang, Chiang Mai	PMNN016	30^a^	15II	2.077 ± 0.007		2×	61.255 ± 1.900		46.719 ± 1.956	
	Doi Suthep, Mueang, Chiang Mai	PMNN017	30^a^	15II	2.093 ± 0.006		2×	60.978 ± 2.144		46.679 ± 2.234	
	Nam Nao-Lom Sak, Phetchabun	PMNN025			2.077 ± 0.009		2×				
	Phan, Chiang Rai	PMNN022	30^a^	15II	2.127 ± 0.018		2×	59.166 ± 3.382		41.793 ± 1.901	
	Rong Kwang, Phrae	PMNN028			2.087 ± 0.005		2×				
	Song, Phrae	PMNN005						59.660 ± 3.750		46.385 ± 2.781	
	Thong Pha Phum, Kanchanaburi	PMNN008	30^a^	15II	2.065 ± 0.019		2×	56.651 ± 3.065		45.806 ± 2.181	
	Wiang Pa Pao, Chiang Rai	PMNN004			2.097 ± 0.033		2×	59.957 ± 3.378		47.471 ± 2.657	
*G. harmandii* (Baill.) K. Schum.	Ban Na, Nakhon Nayok	PMNN024			2.201 ± 0.002	2.195 ± 0.025	2×	69.713 ± 3.807	74.506 ± 5.075	49.621 ± 2.783	56.082 ± 6.459
	Bo Thong, Chonburi	PMNN006	30	15II	2.212 ± 0.031		2×	80.575 ± 4.122		65.625 ± 3.253	
	Khao Chamao, Rayong	PMNN015			2.177 ± 0.014		2×	73.447 ± 2.387		54.881 ± 2.248	
	Mueang Saraburi, Saraburi	PMNN010	30^a^	15II	2.186 ± 0.021		2×	74.289 ± 2.238		54.201 ± 2.355	
*Gagnepainia* sp.	Khong Chiam, Ubon Ratchathani	PMNN021			1.986 ± 0.035	1.986 ± 0.035	2×				

II bivalent, pg picogram, S.E. = standard error, PMNN = P. Moonkaew and N. Nopporncharoenkul ^a^ somatic chromosome number (2*n*) referred from meiotic figure

### Meiotic chromosome analysis

Meiotic configuration was determined using the aceto-orcein smear and DAPI staining techniques with minor modifications, according to the protocols of Nopporncharoenkul et al. (2017) and Mandáková and Lysak (2016). The young inflorescences, with the majority of closed flower buds of each accession, were harvested in the fields or cultivation at their early emerging stage. To stop all cellular activities and reactions, samples were immediately fixed in Carnoy’s fixative (6: 3: 1 v/v of ethanol: chloroform: glacial acetic acid) at room temperature for 24 hours. The fixed flower buds were then transferred and preserved in 70% ethanol at 4 °C until used. Afterwards, selected inflorescences were washed with distilled water twice for 10 min. Each anther, containing the two thecae, was separated from unwanted parts of the closed flower under a stereomicroscope. Finally, a bilocular anther was gently washed with 45% acetic acid at room temperature for 5 min. For individual anther, each theca was used for preparation of meiotic chromosome via either conventional aceto-orcein or fluorescence DAPI (4', 6-diamidino-2-phenylindole) staining.

To further analyze samples, the conventional technique was performed. Anther theca was stained with 1% (w/v) aceto-orcein, and microscopic slide was rapidly moved above the flame of an alcohol burner for three to five times. Warm anther suspension was gently smeared using dissecting needles, and the remaining tissue debris was discarded. A fine cell suspension was covered with a coverslip and tapped vertically with dissecting needles to squash the cells flat. The chromosomes were investigated under an Olympus CX21 light microscope.

If the theca staining with aceto-orcein provided the meiotic chromosomes at the right stage, another half theca would be investigated using fluorescence DAPI staining. The theca was placed on an acid-cleaned microscope slide and treated with 10 μl of 45% (v/v) acetic acid. The anther theca was gently tapped with sterile dissecting needles, and the remaining tissue debris was discarded. A fine cell suspension was covered with 18 × 18 mm coverslip, and tapped vertically with dissecting needles to squash the cells flat. The slide was dipped into liquid nitrogen for 5 seconds, and a coverslip was immediately flicked off with a razor blade. The cells on an air-dried slide were stained with 16 µl of fluorochrome DAPI and covered with 22 × 22 mm coverslip. The chromosomes were investigated under an Olympus BX50 epifluorescent microscope connected to a UV source.

Meiotic figures were determined from the pattern of chromosome pairing during late prophase I to anaphase I at 1000× magnification under an Olympus BX50 epifluorescent microscope. The spread chromosomes were captured with an Olympus DP73 digital camera. Cytogenetic characters of each accession were analyzed from at least 20 cells per plant and three plants per accession.

### DNA content estimation

The nuclear DNA content (2C-value) was estimated using propidium iodide flow cytometry according to the two-step protocol described by Doležel et al. (2007) with minor modifications. *Musa serpentina* Swangpol & Somana, 2011 clone SS&JS 246 with 2C-value = 1.36 pg was used as the internal reference standard (Rotchanapreeda et al. 2016, Nopporncharoenkul et al. 2017). The young leaves of *Gagnepainia* sample and *M. serpentina* were chopped together using a new sharp razor blade in 1 ml of fresh ice-cold nuclei isolation Otto’s buffer I (0.1 M citric acid and 0.5 % Tween 20). The nuclear suspension was filtered through a 42-µm nylon mesh and then centrifuged at 3,500 rpm for 5 min. The supernatant was immediately removed, and the nuclear pellet was then resuspended in 200 µl of ice-cold Otto I solution. After that, 400 µl of Otto II solution (0.4 M Na_2_HPO_4•_12H_2_0 supplemented with 50 µg/ml of propidium iodide (PI), 50 µg/ml of RNase A and 2 µl/ml of β-mercaptoethanol) was applied into the same vial with the nuclear suspension in Otto’s buffer I. The nuclear suspension was incubated for about 30 min at room temperature and then analyzed by BD FACSCallibur Flow Cytometer (BD Biosciences, United States). All histograms were analyzed and gated using the BD FACSDiva version 6.1.1 software (BD Biosciences, United States). Each individual plant was re-analyzed for three times on different days and the final nuclear DNA content of each accession was estimated from three individual plants. The genome size (2C-value) of each *Gagnepainia* accession was calculated by sample G0/G1 mean peak divided by reference standard G0/G1 mean peak and multiplied with reference standard 2C-value (1.36 pg).

### Pollen morphological study

The fresh pollen grains of 13 accessions were directly collected from anthers of the flowers at anthesis stage, and then preserved in 70% ethanol. Six accessions, including PMNN021, 025, 027, 028, 029, and 030, were excluded from this analysis since we could not collect the pollen samples when they were flowering. The hundred grains from individual plant and three plants of each accession were randomly selected for pollen morphological investigation. Pollen unit, shape, size (polar and equatorial axes), aperture, wall thickness and sculpturing were observed and measured using LM and SEM. For SEM investigation, the samples were dehydrated using an ethanol series 70%, 80%, 95%, and 100%, each step for 5 min. The dehydrated pollens were dried in the air at room temperature for overnight and mounted on an aluminium panel affixed to stubs with carbon tape. Consequently, the stubs were sputter-coated with platinum-palladium in a Hitachi E102 ion sputter for 10 min. The pollen morphology was examined and photographed using a Hitachi SU8010 scanning electron microscope at 5 kV. Pollen terminology, according to an illustrated handbook, was used to describe the pollen features (Hesse et al. 2009).

### Statistical analysis

The datasets of pollen sizes (polar and equatorial axes) and nuclear DNA content were initially tested the normal distribution. Analysis of variance (one-way ANOVA) was also conducted using IBM SPSS Statistics version 21.0 software (IBM, United States).

## Results

### Cytogenetic analyses: chromosomes and genome sizes

We successfully obtained meiotic figures from six and two accessions of *Gagnepainia godefroyi* and *G. harmandii* respectively, whereas other accessions did not contain the cells at the right stage for meiotic chromosome study. Only some accessions of *G. godefroyi* provided a fair quality with low contrast of stained chromosomes when using the conventional aceto-orcein staining method. On the other hand, this technique cannot effectively differentiate the chromosomes from cytoplasm in all cases of *G. harmandii* accessions. Therefore, we need to apply the chromosome-specific DAPI fluorochrome staining for clear demarcation of chromosomes in *Gagnepainia*.

**Figure 2.**
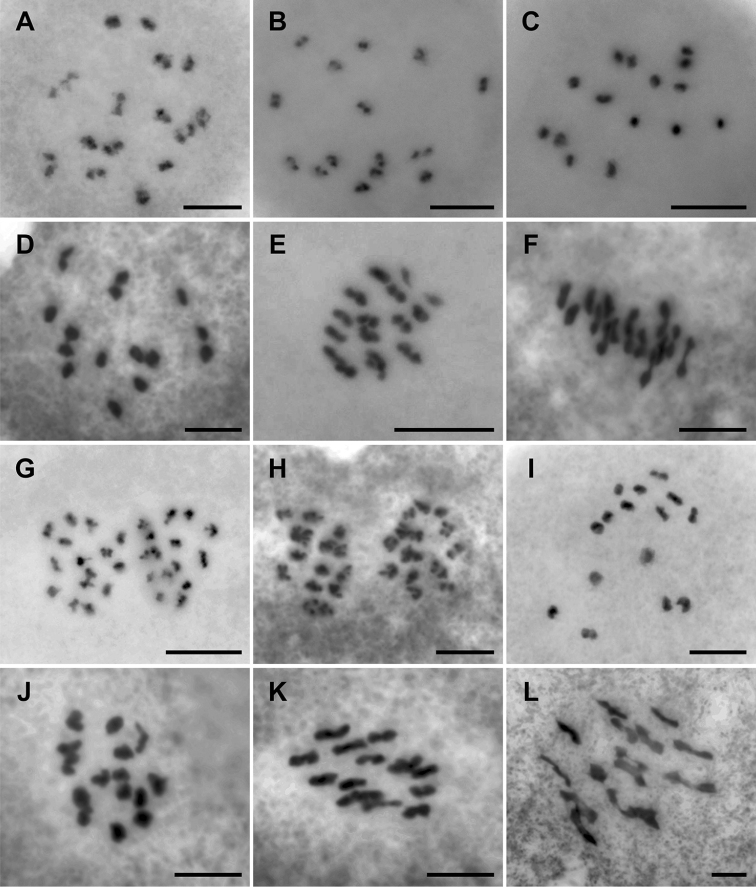
Meiotic chromosomes of *G. godefroyi* (**A–H**) and *G. harmandii* (**I–L**). **A–D** diakinesis **A** PMNN022 **B, C** PMNN017 **D** PMNN008 **E, F** metaphase I **E** PMNN022 **F** PMNN008 **G, H** metaphase I **G** PMNN022 **H** PMNN008 **I, J** diakinesis **I** PMNN006 **J** PMNN010 **K, L** metaphase I, PMNN010. Scale bars: 10 μm.

Results from the meiotic analyses of *Gagnepainia* are shown in Figure 2 and summarized in Table 1. The regular meiotic configuration with the 15 pairs of homologous chromosomes or 15 bivalents (15II) is precisely determined during late prophase I to metaphase I in both *G. godefroyi* and *G. harmandii* (Fig. 2). During the anaphase I, the obvious fifteen homologs are completely separated from equatorial plate to each polar, strongly indicates that the species of *Gagnepainia* are diploid and contain the chromosome number of 2*n* = 2*x* = 30 (Figs 2G, H). The chromosome number of the species of *Gagnepainia*, both mitotic and meiotic, is revealed and reported here for the first time.

**Figure 3.**
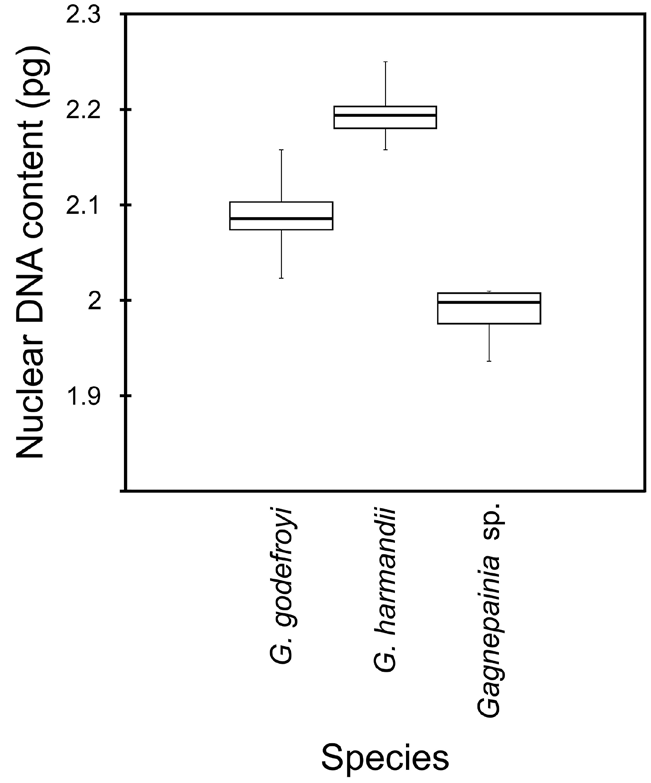
Histograms of the nuclear DNA content of *G. godefroyi* and *G. harmandii*. *Musa serpentina* clone SS&JS 246 was used as the internal reference standard (2C-value = 1.36 pg; Rotchanapreeda et al. 2016).

The genome sizes of the species of *Gagnepainia* were estimated in nuclear DNA content or 2C-value via flow cytometry, compared with the internal standard reference *M. serpentina* clone SS&JS 246 (2C-value = 1.36 pg, Rotchanapreeda et al. 2016). We calculated the nuclear DNA content for the experiments which produced CV < 5%. The mean 2C-values with standard deviation of each species, including *G. godefroyi*, *G. harmandii*, and *Gagnepainia* sp., are 2.090 ± 0.028 (ranges 2.059–2.134), 2.195 ± 0.025 (ranges 2.177–2.212), and 1.986 ± 0.035 pg, respectively (Fig. 3; Table 1).

### Pollen morphology

**Figure 4.**
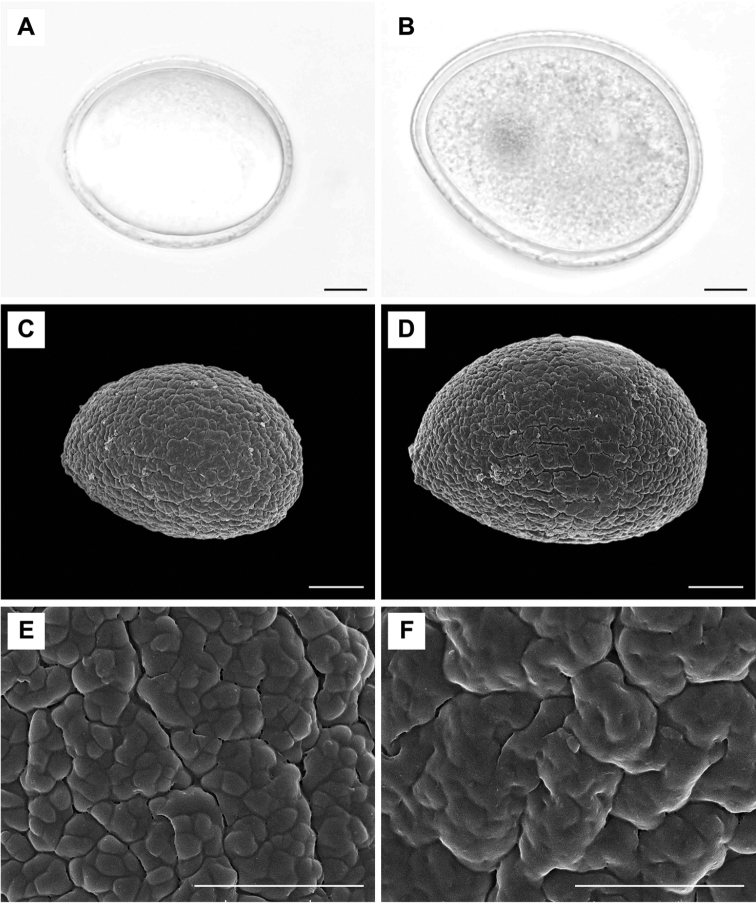
Pollens of *G. godefroyi*, *G. harmandii*, and *Gagnepainia* sp. **A, B** Pollen grains under LM**A***G. godefroyi* PMNN008 **B***G. harmandii* PMNN006 **C, D** Pollen grains under SEM**C***G. godefroyi* PMNN008 **D***G. harmandii* PMNN006 **E, F** Exine sculpturing under SEM**E***G. godefroyi* PMNN008 **F***G. harmandii* PMNN006. Scale bar: 10 μm (**A–D**) and 5 μm (**E, F**).

The pollen characters of *Gagnepainia godefroyi* and *G. harmandii* represent the same pattern in both LM and SEM analyses (Fig. 4; Table 1). The pollen grains are monad, inaperturate, prolate with bilateral symmetry. Pollen sizes of *G. godefroyi* and *G. harmandii* range from 56.651–61.875 and 69.713–80.575 µm in polar axis and 41.793–47.471 and 49.621–65.625 µm in equatorial axis, respectively. The mean pollen size of *G. harmandii* (74.506 ± 5.075, 56.082 ± 6.459) is significantly larger than that of *G. godefroyi* (59.968 ± 3.484, 45.439 ± 2.870). Pollen wall thickness varies from 1.99 to 3.95 to 3.16 to 5.25 µm in *G. godefroyi* and *G. harmandii*, respectively. The pollen exine of the species of *Gagnepainia* has fossulate sculpturing (Fig. 4).

## Discussion

In this study, meiotic chromosomes of the species belonging to *Gagnepainia* are intensively investigated from the young inflorescences with the majority of closed flower buds. Each theca from the same anther is separated and examined cytogenetically using either the conventional aceto-orcein smear, or fluorescence DAPI staining techniques. Unfortunately, the conventional technique with aceto-orcein staining provides the undesirable results of an ambiguous contrast between chromosomes and cytoplasm in both *G. godefroyi* and *G. harmandii*. Because of these results, we precisely examined cytogenetically from another theca using the chromosome-specific DAPI fluorochrome application whenever the nuclei in the cells from the first half theca could not be distinguished using the prior conventional method.

During the microsporogenesis, the regular meiosis with 15 bivalents (15 II) clearly occurs at diakinesis of late prophase I. Moreover, the obvious 30 individual chromosomes during anaphase I are completely separated into 2 sets of 15 chromosomes and moved to each pole in both *Gagnepainia* species analyzed. This meiotic and other evidence, especially the numerous viable seeds found in both natural habitats and in cultivation, strongly indicate that the species of *Gagnepainia* are diploid and have the chromosome number of 2*n* = 2*x* = 30 (Fig. 2; Table 1). As a result, the basic chromosome number of the genus *Gagnepainia* is *x* = 15. When comparing with the genome size of *Musa serpentina* clone SS&JS 246 (Rotchanapreeda et al. 2016), the range of the nuclear DNA content is found ranging from 1.986 pg in *Gagnepainia* sp. accession PMNN021 to 2.212 pg in *G. harmandii* accession PMNN006 (Fig. 3; Table 1). Although both *Gagnepainia* species contain the same chromosome number of 2*n* = 30, they display the different 2C/2*n* values or average chromosome sizes. To conclude, these characteristics, genome sizes, and 2C/2*n* values are slightly different and able to distinguish between two *Gagnepainia* species.

The monoploid genomes (1C*x*-value) of the genera, belonging to the Ginger family, were classified as very small genome which are less than 3.5 pg (Soltis et al. 2003, Leong-Škorničková et al. 2007, Chandrmai et al. 2012, Sadhu et al. 2016, Basak et al. 2018), such as *Alpinia* Roxburgh, 1810 (0.965–1.108 pg), *Curcuma* Linnaeus, 1753 (0.265–0.473 pg), *Globba* (0.750–0.908 pg), *Hedychium* J. Koenig, 1779 (0.678–1.070 pg), *Kaempferia* Linnaeus, 1753 (1.180–1.863 pg), and *Zingiber* Miller, 1754 (1.800–1.945 pg). However, the 1C*x*-value of *Gagnepainia* (0.993–1.106 pg) is closely related to *Globba*, another genus within the same tribe Globbeae.

According to the previous zingiberaceous chromosome reports, the chromosome number of 2*n* = 30 is uniquely found only in tribe Globbeae, especially genus *Hemiorchis* (Pospíšilová et al. 2016). The base chromosome numbers of the family Zingiberaceae have been reported ranging from *x* = 6 to *x* = 25 (Ramachandran 1969, Jatoi et al. 2007). Interestingly, the basic chromosome number *x* = 15 of *Gagnepainia* is a new number in the Ginger family. The chromosome and genome size evidences of the present study fully support the classical taxonomic and phylogenetic classification of Globbeae that *Gagnepainia* is more closely related to *Hemiorchis* than *Globba* (Williams et al. 2004, Pospíšilová et al. 2016). However, *Gagnepainia* and *Hemiorchis* are recognized as distinct genera by use of morphological characters, such as filament length, specific labellum shape, rhizome and tuberous root form (Williams et al. 2004, Picheansoonthon and Tiyaworanant 2010, Pospíšilová et al. 2016).

A monad, inaperturate, prolate with bilateral symmetry, and thick wall with fossulate exine sculptured pollen is recognized as the species of *Gagnepainia* (Fig. 4). The pollen shape and aperture of *Gagnepainia* are similar to those in some genera in Zingiberaceae, especially *Curcuma* and *Hedychium* (Sakhanokho and Rajasekaran 2010, Chen and Xia 2011, Saensouk et al. 2015). However, the nearly smooth pollen exine ornamentation of *Gagnepainia* is obviously different from the echinate exine of *Globba* (Syamsuardi et al. 2010). Amongst the quantitative pollen results, sizes of pollen grains are significant difference between two *Gagnepainia* species, *G. godefroyi* (59.968 ± 3.484 μm, 45.439 ± 2.870 μm) *vs G. harmandii* (74.506 ± 5.075 μm, 56.082 ± 6.459 μm). Therefore, the pollen size is considered as the effective character state that has the potential for species discrimination of *Gagnepainia*.

The high genetic stability with regular meiosis, normal pollen production through producing of numerous viable seeds in natural habitats and cultivation obviously indicates that species of *Gagnepainia* have high fertility and productivity. Theoretically speaking, both *Gagnepainia* species should be broadly distributed in large populations. According to our field studies, these reproductive evidences are in full accordance with previous study that the species of *Gagnepainia* are widely distributed across Indo-China and Thailand, except only the peninsular region which unusually has a high monsoon rainfall (Chuakul and Boonpleng 2004, Techaprasan et al. 2010, Promtep et al. 2011, Leong-Škorničková and Newman 2015, Saensouk et al. 2016). Nevertheless, *G. harmandii* has a more restricted distribution around Central and Eastern Thailand than *G. godefroyi*. This might be caused by the habitat destruction and fragmentation through the expansion of human population and plantation. According to fertility and distribution information, the least concern (LC) has recently been assigned as the IUCN conservation status for both species of *Gagnepainia* (Newman and Simon 2017). However, the field observations of these plants are extremely difficult because they have short flowering period, flowers and leaves usually occur at the different time, and plants mostly grow up in the deep forest coexisting with many zingiberaceous plants. Likewise, in several plants of the Zingiberaceae, the lack of the reproductive parts, especially flowers at anthesis, contributes to easily taxonomic misidentification (Nopporncharoenkul et al. 2017).

On the other hand, *Gagnepainia* sp. accession PMNN021 collected from Khong Chiam, Ubon Ratchathani has distinctive swollen rhizomes jointed with the base of the well-developed pseudostem, which is a unique character, only occurring in Gagnepainia. Since the sample collection, this plant has not produced the inflorescences and flowers. After the genome size has been estimated, this accession contains a significantly different genome size from *G. godefroyi and G. harmandii*. Consequently, this accession may be either a variation of two recognized species, new record, or new species, not identifiable until its flower is intensively observed.

## Conclusion

We have provided beneficial information on the cytological and reproductive aspects of the species belonging to *Gagnepainia*. First of all, the chromosome number *2n* = 30 with the base number *x* = 15 of the genus *Gagnepainia* is revealed here for the first time and recognized as the new number for the Ginger family. Secondly, the genome and pollen sizes in the present study can be used as the effective characteristics for species discrimination between *G. godefroyi* and *G. harmandii*. This is especially useful as both species of *Gagnepainia* have herbal properties used for treatment of wounds and inflammations, and also have the numerous attractive butterfly-like flowers. Thirdly, they have the high potential to be developed as the commercial medicinal and ornamental pot plants through breeding and genetic improvement programs, such as polyploid induction. Fourthly, the present cytogenetic study has provided not only informative characteristic for species discrimination, but also very useful assessment for plant fertility through *in situ* and *ex situ* conservation strategies, plant genetic resource management, and plant improvement programs. Last but not least, karyotyping of mitotic chromosomes and application of fluorescence in situ hybridization (FISH) should be investigated in future research in order to precisely understand chromosomal evolution between the genera *Gagnepainia* and *Hemiorchis*.
